# RON Mediates Tumor-Promoting Effects in Endometrial Adenocarcinoma

**DOI:** 10.1155/2021/2282916

**Published:** 2021-10-19

**Authors:** Qin Yu, Jianzhang Wang, Tiantian Li, Xinxin Xu, Xinyue Guo, Shaojie Ding, Libo Zhu, Gen Zou, Yichen Chen, Xinmei Zhang

**Affiliations:** ^1^Department of Obstetrics and Gynecology, Women's Hospital, School of Medicine, Zhejiang University, Hangzhou, 310006 Zhejiang, China; ^2^Ningbo Institution of Medical Science, Ningbo, 315000 Zhejiang, China

## Abstract

Endometrial adenocarcinoma is one of the most prevalent female reproductive tract cancers in the world, and the development of effective treatment is still the main goal of its current research. Epithelial-mesenchymal transition (EMT) plays a significant part in the occurrence and development of epithelial carcinoma, including endometrial adenocarcinoma. Recepteur d'origine nantais (RON) induces EMT and promotes proliferation, migration, and invasion in various epithelial-derived cancers, but its role in endometrial adenocarcinoma is still poorly studied. The purpose of this study is to verify the overexpression of RON in endometrial adenocarcinoma and to explore its specific roles. RON expression in tumor lesions was verified by immunohistochemical staining, HEC-1B cells were used to construct stable cell lines with RON overexpression or knockdown to investigate the effects of RON on the function of endometrial adenocarcinoma cells, and xenotransplantation experiment was carried out in nude mice to explore the effect of RON on the growth of endometrial adenocarcinoma *in vivo*. This study revealed that RON could promote the proliferation, migration, and invasion of HEC-1B cells and induce EMT, and these effects were regulated through the Smad pathway. RON overexpression could promote growth of endometrial adenocarcinoma cells in nude mice, while its inhibitor BMS777607 could restrict this role. RON played an important role in endometrial adenocarcinoma and had a potential to become a new therapeutic target for endometrial adenocarcinoma.

## 1. Introduction

Endometrial carcinoma is a common malignancy of female reproductive tract worldwide, and its incidence is increasing gradually. About 80% of endometrial cancer cases are estrogen-dependent type I endometrial cancer, also known as endometrial adenocarcinoma [1, 2]. Although endometrial adenocarcinoma usually has a better prognosis, 13-25% of patients still suffer from recurrence and metastasis [3, 4]. The metastasis and spread of cancer are usually the main cause of poor prognosis [5, 6], in which epithelial-mesenchymal transition (EMT) plays a vital role [7, 8]. EMT describes a biological process by which epithelial cells lose their identities and gain power to become spindle-shaped mesenchymal cells [9, 10]. EMT is involved in important biological processes such as embryogenesis, wound healing, and tissue regeneration [11, 12]. Numerous studies have shown that EMT is reactivated in malignant tumors of epithelial origin, enabling tumor cells acquire characteristics of stem cells and promoting the onset and progression of tumors [13–15]. E-cadherin expression was found to be reduced in endometrial adenocarcinoma tissues [16, 17], suggesting that EMT is related in the occurrence and development of endometrial adenocarcinoma, and EMT can be used as an entry point for research.

Tyrosine kinase receptor Recepteur d'origine nantais (RON), discovered in 1993, has been extensively studied for its overexpression in a variety of epithelial-derived tumors [18–20]. In epithelial carcinoma, RON overexpression is considered to be an important factor for metastasis and poor prognosis [21, 22]. Abnormal expression of RON could induce EMT of tumor cells, promote migration, invasion and proliferation, etc., and thereby inhibition of RON can reverse these characteristics [23–25]. Therefore, targeting RON has become a promising cancer treatment strategy. Although the role of RON has been extensively studied in various types of cancer, the study of RON in endometrial adenocarcinoma is few. Zhuang et al. used real-time polymerase chain reaction (RT-PCR) to detect endometrial adenocarcinoma tissues and found that RON was overexpressed in endometrial adenocarcinoma tissues, and RON expression is associated with histological stage, muscle invasion, and lymph node metastasis [26], but the exact mechanism is unclear.

In this study, we first verified RON was overexpressed in endometrial adenocarcinoma tissues and then constructed HEC-1B cells with RON overexpression or knockdown to verify whether RON acts on endometrial adenocarcinoma cells through EMT. Finally, the role of RON in endometrial adenocarcinoma *in vivo* and the therapeutic effect of RON inhibitor BMS777607 were confirmed by xenograft test in nude mice.

## 2. Materials and Methods

### 2.1. Samples and Immunohistochemical (IHC) Staining

With the approval of the Human Ethics Committee of Women's Hospital, School of Medicine, Zhejiang University (approval number: IRB-20210050-R), we collected 38 paraffin specimens of endometria from 2018 to 2019, including 19 cases of endometrial adenocarcinoma and 19 cases of endometria from patients undergoing endometrial curettage for gynecological benign diseases served as control. After paraffin sectioning, dewaxing, antigen repair, and other steps, the slices were incubated with anti-RON (1 : 500; ab52927; Abcam, Cambridge, Cambridgeshire, UK) antibody at 4°C overnight. Then, the slices were incubated with secondary antibody, stained with DAB and hematoxylin, and next sealed with coverslips. IHC scoring was similar to the previous studies [27, 28]. The stained sections were analyzed and scored by two researchers who were unaware of the clinical data. Five fields were selected for each section, and the staining intensity and percentage were scored. The sum of the two scores was the IHC score of the slice. The staining intensity was divided into 0 (no staining), 1 (weak staining), 2 (moderate staining), and 3 (strong staining). The staining distribution score was 0 (no standing), 1 (1–25%), 2 (25–50%), and 3 (50–100%).

### 2.2. Human Endometrial Adenocarcinoma Cell Culture and Intervention

Human endometrial adenocarcinoma cells (HEC-1B cells) were purchased from CBTCCCAS (Cell Bank, Type Culture Collection, Chinese Academy of Sciences) (catalog number: TCHu115) and then cultured in Minimum Essential Medium (MEM) containing 10% (*v*/*v*) fetal bovine serum (FBS), 50 U/mL penicillin, and 50 *μ*g/mL streptomycin. Cells were serum starved in culture medium with 0.5% FBS for 24 h before treatment with BMS777607 (Selleck, Munich, Germany).

### 2.3. Cell Proliferation Assay

The proliferation ability of HEC-1B cells was investigated by CCK-8 assay. 1 × 10^4^ cells were placed in each well of 96-well plate and incubated at 37°C; then, CCK-8 reagent (Dojindo, Kumamoto, Kumamoto Prefecture, Japan) was added at 0, 24, 48, 72, and 96 h, respectively. After continuous culture for 1 h, Varioskan® Flash (Thermo Fisher Scientific, Waltham, MA, USA) was used for detecting the absorbance of the cells at 450 nm. This experiment was repeated three times.

### 2.4. Migration and Invasion Assays

5 × 10^4^ cells were dispersed in 200 *μ*L MEM without FBS and placed in the upper chamber of 24 well Transwell™ plates (8 *μ*m pore size; Corning, New York City, New York, USA). Then, the lower chamber was added 500 *μ*L MEM containing 10% FBS. After incubating at 37°C for 8 h, the cells were fixed and stained using crystal violet solution containing ethanol and then wash off the unmigrated cells. Five visual fields were selected for each well to count and take photos. For invasion experiment, the upper chamber was incubated at 37°C for 1 h with Matrigel™ (#356234; BD Biosciences, Franklin Lakes, NJ, USA), and then, the experiment was carried out according to the above steps. Three times were repeated in this experiment.

### 2.5. Real-Time Quantitative Polymerase Chain Reaction (RT-qPCR)

Under the guidance of the instructions, mRNA was extracted using RNA-Quick Purification Kit (RN001, Shanghai Yishan Biotechnology Co., Ltd., Shanghai, China) and then reverse transcribed into cDNA using PrimeScript™ Reverse Transcription Reagent kit (TaKaRa Biotechnology, Shiga, Japan). RT-PCR was performed using SYBR Premix Ex Taq Kit (Takara Biotechnology). Primers for amplification are listed in Table 1. Experiment was repeated three times, and the relative expression was calculated by 2^–*ΔΔ*CT^ method.

### 2.6. Western Blotting

Protein samples were prepared by lysing cells using RIPA lysis buffer (including PMSF, Beyotime Institute of Biotechnology, Shanghai, China), and the concentration was detected by BCA protein detection kit (#23227; Thermo, Waltham, MA, USA). Then, protein samples were added into SDS-PAGE gel; after electrophoresis separation, they were transferred to PVDF membrane. After blocking with 5% bovine serum albumin at room temperature for 1 h, the membrane was incubated with primary antibody at 4°C overnight. The antibodies used are as follows: ZEB2 (1 : 1000; sc-271984; Santa Cruz Technology, CA, USA), RON *β* (1 : 500; sc-25781; Santa Cruz Technology), E-cadherin (1 : 1000; 3195S; Cell Signaling Technology), Vimentin (1 : 1000; 5741S; Cell Signaling Technology), SMAD1/5/9 (1 : 1000; AF0614; Affinity Biosciences), P-SMAD1/5/9 (1 : 1000; 13820S; Cell Signaling Technology), and GAPDH (1 : 1000; A95370123; Multisciences Biotech, Shanghai, China). On the second day, the corresponding mouse (1 : 10 000, ab97023, Abcam) or rabbit (1 : 10 000, ab97051, Abcam) secondary antibodies were incubated for 1 h at room temperature after membrane cleaning. After cleaning again, FDbio-Dura ECL Kit (FDbio Science Biotech Co., Ltd., Hangzhou, Zhejiang, China) was used to detect the bands, and the results were analyzed with the ImageJ software (National Institutes of Health, Bethesda, MD, USA). Three times were repeated in this experiment.

### 2.7. Establishment of Stable RON Knockdown or Overexpression Cell Lines

In order to establish stable RON knockdown cells (shRON), shRNA (gctggctctcattggtatcatctcgagatgataccaatgagagccagctttttt) was inserted into pLVX-shRNA-Puro-ZsGreen1vector (GeneCreate Biological Engineering Co., Ltd., Wuhan, Hubei, China). Besides, full-length human RON cDNA was amplified and subcloned into GV492 lentiviral vector (Genechem Co., Ltd., Shanghai, China) to build up stable RON overexpression cell line (RON-OE). Then, lentivirus packaging, cell infection, and screening for stable transfection cell lines were carried out pursuant to the manufacturers' instructions. Their corresponding control cells (shCtrl and NC) were obtained using the same vector without nucleotide sequence.

### 2.8. Animals and Treatment

Twenty-four 4-5 weeks old female BALB/C nude mice were purchased from Shanghai Animal Center (Chinese Academy of Science, Shanghai, China). After 1 week of acclimation, these mice were distributed into four groups; the first group was injected with shCtrl cells (*n* = 4), the second group was injected with shRON cells (*n* = 4), the third group was injected with NC cells (*n* = 4), and the last group was injected with RON-OE cells (*n* = 12). 1 × 10^6^ of the above cells was injected into subcutaneous tissue of the right axilla of mice. On the first day after injection of tumor cells, the mice injected RON-OE cells were divided into three groups: one was control group (RON-OE group), another group treated with 15 mg/kg BMS777607 (RON-OE + BMS 15 mg/kg group), and the other group treated with 30 mg/kg BMS777607 (RON-OE + BMS 30 mg/kg group). In the following 21 days, the NC group and RON-OE group were treated with blank solvent (dimethyl sulfoxide: polyethylene glycol 400: water = 3 : 70 : 27) by intragastrical administration every day, and RON-OE + BMS 15 mg/kg group and RON-OE + BMS 30 mg/kg group were treated with BMS777607 according to the corresponding dosage. Vernier caliper was used to measure tumor size every 3 days, and the volume was calculated as *V* = 0.5 × length × width^2^. Our experimental scheme has been approved by the ethics committee of Zhejiang University (approval number: ZJU20210048).

### 2.9. Data Analysis

The SPSS 17.0 software (IBM, Armonk, NY, USA) was used for statistical analysis. Shapiro-Wilk test was used to verify the normality of the data. Then, student *t*-test (two-tailed) was performed for the two groups of data conforming to normal distribution, and one-way ANOVA test was performed for the three groups or more. For the two groups of data with nonnormal distribution, the Mann–Whitney test was used to analyze their differences. *P* < 0.05 was considered to be significant. The GraphPad Prism 7 software (GraphPad Software, California, USA) was used for graphing, and the results were expressed as mean ± SD.

## 3. Results

### 3.1. RON Was Overexpressed in Endometrial Adenocarcinoma

**Figure 1:**
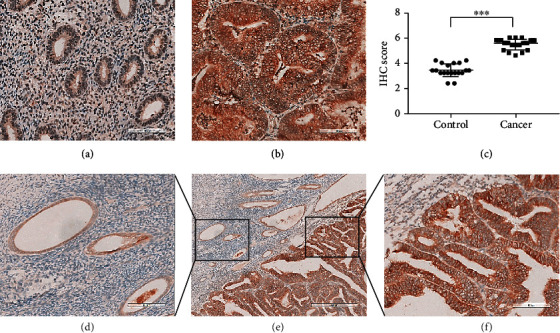
RON was overexpressed in endometrial adenocarcinoma. (a) RON staining on control endometria. (b) RON staining on endometrial adenocarcinoma tissues. Scale bar is 100 *μ*m. (c) Statistical results of immunohistochemical score of RON staining. The values represent the mean ± SD, ^∗∗∗^*P* < 0.001. Adjacent noncancerous tissues (d) (scale bar = 100 *μ*m) and adenocarcinoma endometria (f) (scale bar = 100 *μ*m) from the same patient with endometrial adenocarcinoma ((e) scale bar = 400 *μ*m).

We performed IHC staining on the control endometria of patients without endometrial cancer (*n* = 19) and endometrial adenocarcinoma tissues (*n* = 19). The results showed that the staining intensity of RON in endometrial adenocarcinoma group was higher than that of the nonendometrial adenocarcinoma group (Figures 1(a)–1(c)). In the same patient, the expression of RON in endometria of cancer tissue was higher than that of adjacent noncancerous tissues (Figures 1(d)–1(f)). These results manifested the overexpression of RON in endometrial adenocarcinoma.

### 3.2. RON Promoted Proliferation, Migration, and Invasion of Endometrial Adenocarcinoma Cells

**Figure 2:**
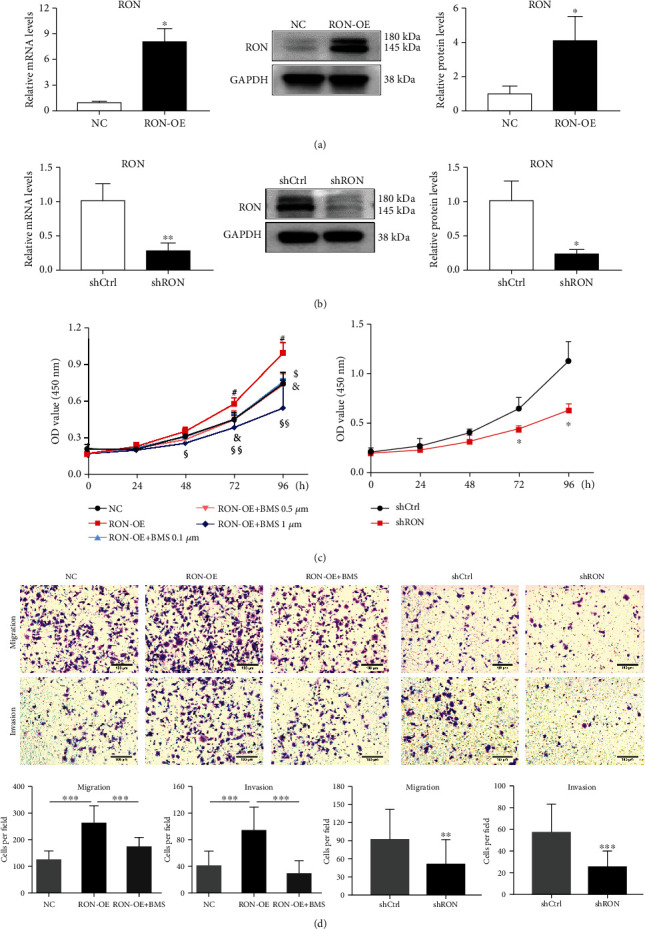
RON promoted proliferation, migration, and invasion of HEC-1B cells. (a) RON expression in HEC-1B cells with RON overexpression (RON-OE). (b) RON expression in HEC-1B cells with RON knockdown (shRON). (c) CCK8 assays of RON-OE and shRON cell lines. #*P* < 0.05, RON-OE group versus NC group; $*P* < 0.05, RON-OE+ BMS 0.1 *μ*M group versus RON-OE group; &*P* < 0.05, RON-OE+ BMS 0.5 *μ*M group versus RON-OE group; §*P* < 0.05, §§*P* < 0.01, RON-OE+ BMS 1 *μ*M group versus RON-OE group. (d) The transwell assays of RON overexpressing or knockdown cells. BMS means that the cells were exposed to BMS777607 (1 *μ*M) for 24 h before transwell assay. The scale bars represent 100 *μ*m. All of assays were repeated three times, and values represent the mean ± SD, ^∗^*P* < 0.05, ^∗∗^*P* < 0.01, ^∗∗∗^*P* < 0.001.

We used HEC-1B cells for following experiments, and we successfully constructed RON overexpression (RON-OE) and knockdown (shRON) cell lines. In RON-OE cells, the mRNA and protein levels of RON increased 8-fold and 4-fold, respectively, compared with the NC cells (Figure 2(a)). In shRON cells, both the mRNA and protein levels of RON decreased by 70% compared with control cells (shCtrl) (Figure 2(b)). We tested the effect of RON on the proliferation of endometrial cells using CCK8 kit, and the results revealed that overexpression of RON could promote the proliferation of HEC-1B cells, and BMS777607 (the inhibitor of RON) could inhibit the proliferation drived by RON. Knockdown of RON could also inhibit the proliferation of HEC-1B cells (Figure 2(c)).

Then, we tested the function of RON on the migration and invasion of HEC-1B cells.  Figure 2(d) shows that overexpression of RON could significantly increase the number of cells passing through the chamber during migration and invasion assay, which was about twice of the control group, while BMS777607 could reverse this effect. Additionally, knockdown of RON in HEC-1B cells could reduce the number of cells passing through by about 50%. The results indicated that RON could promote proliferation, migration, and invasion of endometrial adenocarcinoma cells.

### 3.3. RON Induced EMT of Endometrial Adenocarcinoma Cells

**Figure 3:**
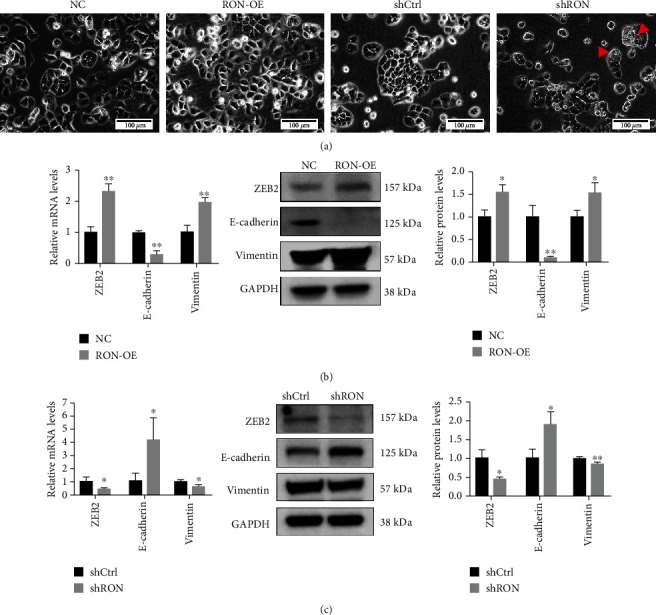
RON induced EMT of HEC-1B cells. (a) The effect of RON overexpression or knockdown on morphological changes of HEC-1B cells. The red arrow marked the vacuoles in shRON cells. The scale bars represent 100 *μ*m. (b) The effect of RON overexpression on the expression of EMT markers in HEC-1B cells. (c) The effect of RON knockdown on the expression of EMT markers in HEC-1B cells. Values represent the mean ± SD, ^∗^*P* < 0.05, ^∗∗^*P* < 0.01. All of assays were repeated three times.

Combined with the above results, RON improved the abilities of HEC-1B cells to proliferate, migrate, and invade, which was related to EMT. So we continued to explore whether RON can cause EMT of HEC-1B cells.  Figure 3(a) shows that RON-OE cells presented a fusiform, fibroblast-like phenotype with reduced intercellular contact and scattered cell clusters, which were all characteristics of EMT. The morphology of shRON cells did not change, and vacuoles could be seen in their cytoplasm (as shown by the arrow in Figure 3(a)), which may also be the reason for the decreased proliferation ability of shRON cells. The results of PCR and Western Blotting showed that overexpression of RON could increase expression of ZEB2 and Vimentin and decrease E-cadherin expression (Figure 3(b)), while the expression of these molecules was reversed when RON was knocked down (Figure 3(c)). All these results indicated that RON played the role in endometrial adenocarcinoma cells by inducing EMT.

### 3.4. Ron Might Play Its Role in Endometrial Adenocarcinoma Cells through Smad Pathway

**Figure 4:**
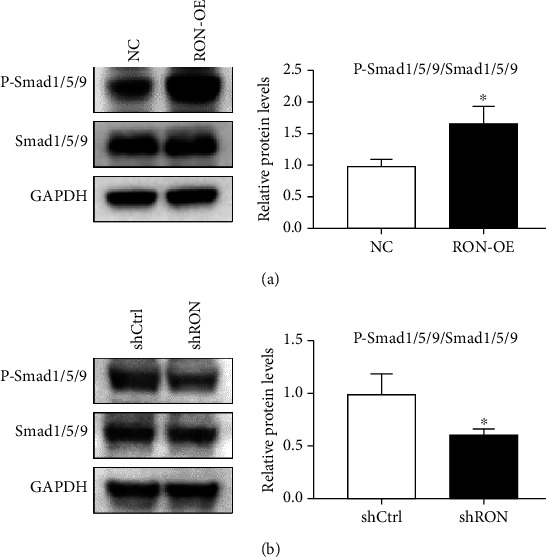
RON might play its role in endometrial adenocarcinoma cells through the Smad pathway. (a) Expression of p-smad1/5/9 in RON-OE cells. (b) Expression of p-smad1/5/9 in shRON cells. Values represent the mean ± SD, ^∗^*P* < 0.05. The assays were repeated three times.

In order to explore the RON-mediated pathway, we examined phosphorylation of pathways in RON overexpression or knockdown cell proteins. We found that the phosphorylation level of Smad1/5/9 increased by about 1.6 times when RON was overexpressed (Figure 4(a)) and decreased by about 40% when RON was knocked down (Figure 4(b)). These results suggested that RON might play its role in HEC-1B cells through Smad pathway.

### 3.5. RON Promoted the Growth of Endometrial Adenocarcinoma in Nude Mice Xenografts

We further investigated the function of RON on the growth of HEC-1B cells in nude mice xenografts. During the postimplantation evaluation period, the diet, activity, and mental status of the nude mice in all groups were normal, and they were all survived during the study period.

**Figure 5:**
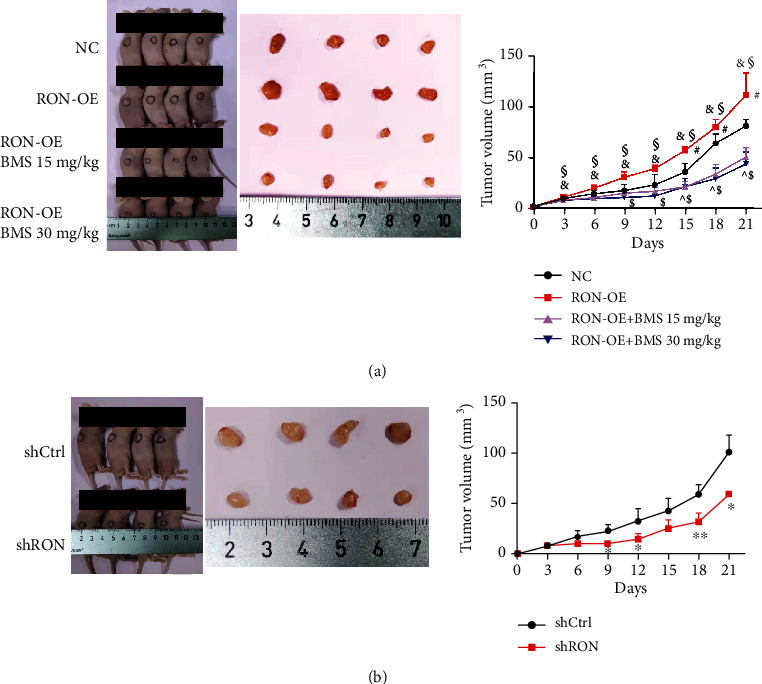
RON promoted the growth of endometrial adenocarcinoma in nude mice xenografts. (a) The effect of RON overexpression on the growth of HEC-1B cells in vivo. #*P* < 0.05, RON-OE group versus NC group; &*P* < 0.05, RON-OE+ BMS 15 mg/kg group versus RON-OE group; §*P* < 0.05, RON-OE+ BMS 30 mg/kg group versus RON-OE group; ^*P* < 0.05, RON-OE+ BMS 15 mg/kg group versus NC group; $*P* < 0.05, RON-OE+ BMS 30 mg/kg group versus NC group. (b) The effect of RON knockdown on the growth of HEC-1B cells in vivo. *n* = 4; values represent the mean ± SD, ^∗^*P* < 0.05, ^∗∗^*P* < 0.01.

Figure 5(a) shows that RON overexpression could promote the growth of endometrial adenocarcinoma in nude mice, which was 38% larger than that in the NC group. BMS777607 inhibited the effect of RON overexpression on tumor growth and reduced tumor size by more than 50%. Compared with control mice, xenograft tumors in nude mice injected with RON knockdown cells grew slowly, and the tumor size was reduced by about 37% (Figure 5(b)). I*n vivo* experiments further showed that RON played a significant part in the development of endometrial adenocarcinoma.

## 4. Discussion

In this research, we investigated the role of RON in endometrial adenocarcinoma. First, we detected the expression of RON by immunohistochemistry and found strong staining in endometrial adenocarcinoma. This proved the high expression of RON in endometrial adenocarcinoma at the protein level, which was similar to the results obtained by Zhuang et al. using RT-PCR to investigate endometrial adenocarcinoma tissues [26]. Subsequently, HEC-1B cells were used to construct RON overexpression or knockdown cells to further explore the effect of RON. We found that RON overexpression significantly increased the proliferation, migration, and invasion ability of HEC-1B cells, while RON knockdown exerted the opposite effect, and RON inhibitor BMS777607 could counteract the effect of RON overexpression. This suggested that RON played a role in promoting proliferation and metastasis in endometrial adenocarcinoma and that was similar to the role of RON played in other cancers [29, 30], and these effects were closely related to EMT.

Then, we explored the effect of RON overexpression or knockdown on the EMT of HEC-1B cells. Previously study has reported that RON activation could induce spindle morphology in MDCK cells [31]; our results found that RON overexpression could also cause spindle morphology of HEC-1B cells and destroy intercellular junctions. The EMT markers in HEC-1B cells affected by RON were E-cadherin, ZEB2, and Vimentin. E-cadherin is an adhesion transmembrane protein between cells, and its loss is considered to be a marker of EMT [32]. It has also been shown to be the key to progression of endometrial cancer, promoting tumor deep invasion of the myometrium [33]. Decreased expression of E-cadherin is usually caused by EMT transcription factors inhibiting its transcription. Common transcription factors include ZEB family, Snail family, or Twist family [34, 35]. In HEC-1B cells, we found that the transcription factor affected by RON was ZEB2, which was consistent with previous results obtained using MSP to stimulate endometrial epithelial cells with MSP (the agonist of RON) [36]. Increased expression of ZEB2 resulted in decreased expression of E-cadherin. RON also induced changes in the expression of Vimentin in HEC-1B cells. Vimentin is mainly expressed in stromal cells; many researches have revealed that its increased expression was associated with invasive cancer, which could promote the migration and invasion of tumor cells [37, 38]. In summary, RON could regulate the EMT of endometrial adenocarcinoma HEC-1B cells. RON generally regulates EMT through the coordinated activation of the RAS-MAPK and PI-3K-Akt pathways [21, 39] and sometimes through the SMAD and JAK pathways [40, 41]. Similar to studies on HK-2 and NRK49F cells from Park et al. [42], our results showed that RON regulated HEC-1B cells through the Smad pathway.

Based on the above results, we confirmed that RON could regulate the proliferation and EMT in endometrial cancer cells, which promoted the development of endometrial cancer. Furthermore, we used xenograft experiments in nude mice to further explore the role of RON. We found that RON promoted the growth of tumor *in vivo*. Concordant with the results obtained in gastric adenocarcinoma GTL16 cells and non-small-cell lung cancer EBC1 cells [43], RON knockdown significantly inhibited the tumor growth of HEC-1B cells in nude mice. We also found that RON inhibitor BMS777607 slowed down the tumor growth of HEC-1B cells caused by RON overexpression.

## 5. Conclusions

RON promotes the proliferation, EMT, migration, and invasion in endometrial adenocarcinoma HEC-1B cells, and these may be regulated through the Smad pathway. RON overexpression could promote the growth of tumor in nude mice xenografts, and BMS777607 could effectively inhibit this effect. Therefore, RON has the potential to become a new therapeutic target for endometrial adenocarcinoma.

## Figures and Tables

**Table 1:** Primers used in real-time PCR.

Gene	Forward primer (5′ to 3′)	Reverse primer (5′ to 3′)
ZEB2	GCGATGGTCATGCAGTCAG	CAGGTGGCAGGTCATTTTCTT
E-cadherin	ATTTTTCCCTCGACACCCGAT	TCCCAGGCGTAGACCAAGA
Vimentin	CCTTGAACGCAAAGTGGAATCT	CCACATCGATTTGGACATGCT
GAPDH	GTCAAGGCTGAGAACGGGAA	AAATGAGCCCCAGCCTTCTC

## Data Availability

The datasets used and/or analyzed during the present study are available from the corresponding author on reasonable request.
